# Evaluation of Physical Properties in Carboxymethyl Chitosan Modified Glass Ionomer Cements and the Effect for Dentin Remineralization: SEM/EDX, Compressive Strength, and Ca/P Ratio

**DOI:** 10.1055/s-0044-1786864

**Published:** 2024-07-16

**Authors:** Aditya Wisnu Putranto, Ratna Meidyawati, Senyan Dwiseptyoga, Dicky Yudha Andhika Zikrullah

**Affiliations:** 1 Department of Conservative Dentistry, Faculty of Dentistry, Universitas Indonesia, Jakarta, Indonesia

**Keywords:** glass ionomer cement, carboxymethyl chitosan, surface morphology, compressive strength, remineralization, dentin morphology, calcium ion, Ca/P

## Abstract

**Objective** The aim of this article was to evaluate the effects of modifying glass ionomer cement (GIC) with carboxymethyl chitosan (CMC) on surface morphology and remineralization outcomes by examining dentin morphology and calcium ion composition changes.

**Materials and Methods** Thirty holes in a cylindrical acrylic mold were filled with three groups of restorative materials: GIC, GIC modified with CMC (GIC-CMC) 5%, and GIC-CMC10%. The surface morphology of each group's materials was observed using scanning electron microscopy (SEM). The compressive strength measurement was performed using a universal testing machine. The dentin remineralization process was performed by applying GIC, GIC-CMC5%, and GIC-CMC10% materials for 14 days on demineralized dentin cavities treated with 17% ethylenediamine tetraacetic acid (EDTA) for 7 days. A morphological evaluation was conducted using SEM. The calcium ion composition and calcium-to-phosphorous (Ca/P) ratio were examined using an energy-dispersive X-ray (EDX).

**Statistical Analysis** The one-way ANOVA and post-hoc Bonferroni test were used to evaluate the compressive strength within the three groups ( *p* < 0.05). The Kruskal–Wallis and subsequent Mann–Whitney U tests were conducted to compare the four groups of calcium ions ( *p* < 0.05).

**Results** The modification of GIC with CMC affected the morphological changes in the materials in the form of reduced porosity and increased fractures. A significant difference was found in compressive strength between the GIC-CMC modification materials of GIC-CMC5% and GIC-CMC10% and the GIC control group. The dentin tubule morphology and surface changes were observed after applying GIC, GIC-CMC5%, and GIC-CMC10% materials for 14 days, as evaluated by SEM. The EDX examination showed an increase in calcium ion content and hydroxyapatite formation (Ca/P ratio) after applying the GIC-CMC10% material.

**Conclusion** The surface porosity of the GIC modification material with the addition of CMC tended to decrease. However, an increase in cracked surfaces that widened, along with the rise in CMC percentage, was found. This modification also reduced the compressive strength of the materials, with the lowest average yield at 10% CMC addition. Therefore, the modification of GIC with CMC affects changes in morphology, calcium ion composition, and Ca/P ratio in demineralized dentin.

## Introduction

Remineralization is necessary to enable minerals to occupy the intrafibrillar spaces within dentin collagen, allowing for the long-term success of dental restoration and caries prevention. 1 2 The concept of returning minerals to dentin, often referred to as remineralization, can be divided into two methods: the classic (top-down) approach and the biomimetic (bottom-up) approach. The classic method, which was discovered earlier and is commonly used today, has a limitation in that it leaves voids within the intrafibrillar collagen of dentin. This is because this method cannot deliver minerals to these locations, only being able to restore minerals to the extrafibrillar collagen of dentin. 3 This unresolved issue can lead to problems regarding the complete treatment of caries, as discussed previously.

Conversely, the more modern concept of biomimetic remineralization seeks to address the issues of dentin caries and classic remineralization by filling both intrafibrillar and extrafibrillar collagen spaces. This approach aims to eliminate any gaps that could disrupt dental restorations or make dentin vulnerable to demineralization again. This concept requires a supply of calcium ions and noncollagenous protein analogs (NCP) to stabilize calcium ions for entry into intrafibrillar collagen. 1 3 4 Carboxymethyl chitosan (CMC) is a naturally derived biocompatible material that can serve as an NCP analog. 5 In earlier biomimetic remineralization investigations, CMC was found to act as a natural substitute for dentin matrix protein 1 (DMP-1) into an NCP sequestration analog that stabilizes amorphous calcium phosphate (ACP) nano precursors so that they do not aggregate into large apatite particles before entering the intrafibrillar collagen compartment, thus helping the bottom-up remineralization process. 5 6 7 Putranto et al stated that the use of novel cement-based CMC/ACP (CMC/ACP modified to gypsum) showed amorphous characteristic, which can stabilize calcium ions and phosphate group (ACP). 8 The addition of CMC to bioactive material that releases calcium ions, such as calcium silicate cement—mineral trioxide aggregate (MTA), has been found to increase the potential for biomimetic remineralization tested by scanning electron microscope (SEM) and energy dispersive X-ray (EDX) analysis that shows increased calcium ion activity as well as dentin remineralization by hydroxyapatite (HAP). 6 Unfortunately, CMC modifications with MTA have weak physical characteristics, so this material is not recommended as a single ingredient for restoring caries teeth. 6

Glass ionomer cement (GIC) is a dental material widely used for restorations, luting, lining, and sealants, and it continuously releases calcium ions. 9 GIC has low physical properties in comparison with other restoration material such as composite resin and is continuously researched to improve its properties by modifying its components. 10 11 The study from Arjomand et al showed that the compressive strength of various GIC increases with aging; however, these GICs fail to perform as efficient as composite resin. 11 Bao et al on their research used GIC-modified material through the addition of CMC powder in a certain amount of mass (0.00625wt.%, 0.0125wt. %, 0.025wt.%, and 0.0375wt.) on the liquid component of GIC, an increase in compression strength on the modified materials. 12 Kashyap et al, in their study, used a modified GIC powder (homogenized in a ball mill) with the addition of carboxymethyl chitosan (10 wt.%) to form a carboxymethyl-chitosan-modified glass powder (CMC-m-GP), improved compressive strength and flexural strength. 13 Studies by Pratiwi et al showed that modifying the GIC using the Xylotrupes gideon nano-chitosan increased the cracks on the surface morphology of GIC. 14 However, the mixing technique for modified GIC and chitosan mentioned by Bao et al. and Kashyap et al. were different compared with Mulder and Anderson-Small using a w/w% ratio and analyzing the ion release that affected remineralization in carious dentin. 12 13 15 The modification of CMC-ACP as scaffolds with various concentration (1%, 2.5%, 5%, and 10%) showed higher calcium and phosphate level when applying 10% CMC/ACP on demineralized dentin. 16

Mulder and Anderson-Small found that the combination of chitosan with GIC enhances the ion release capabilities of GIC. 15 GIC and chitosan have been studied and found to interact effectively due to the hydroxyl groups in chitosan, which can bond with the inorganic materials in GIC. 17 Based on the potential of biomimetic remineralization of these two materials and earlier research, further investigation is required to understand the effects of modifying GIC with CMC in initiating remineralization in demineralized dentin. 12 15 In the this study, this was accomplished by observing the surface morphology of dentin using SEM and analyzing the composition of remineralization ions using EDX examination and continuing with calcium-to-phosphorous (Ca/P) ratio analysis. This study also aims to analyze the surface morphology of GIC modified with carboxymethyl chitosan using SEM and a compressive strength test using a universal testing machine to study the effects of this modification on the properties of the materials. To date, no research has explained how the preparation of CMC in powder with 5 and 10% concentrations can mix with GIC as a cement material suitable for conservative dentistry treatment such as dentin remineralization. The null hypothesis was that the increased concentration of CMC in GIC (1) affects the surface morphology of the materials, (2) the compressive strength of the materials, (3) the morphology of dentin tubules, (4) increases calcium ion, and (5) Ca/P ratio.

## Materials and Methods

**Fig. 1:**
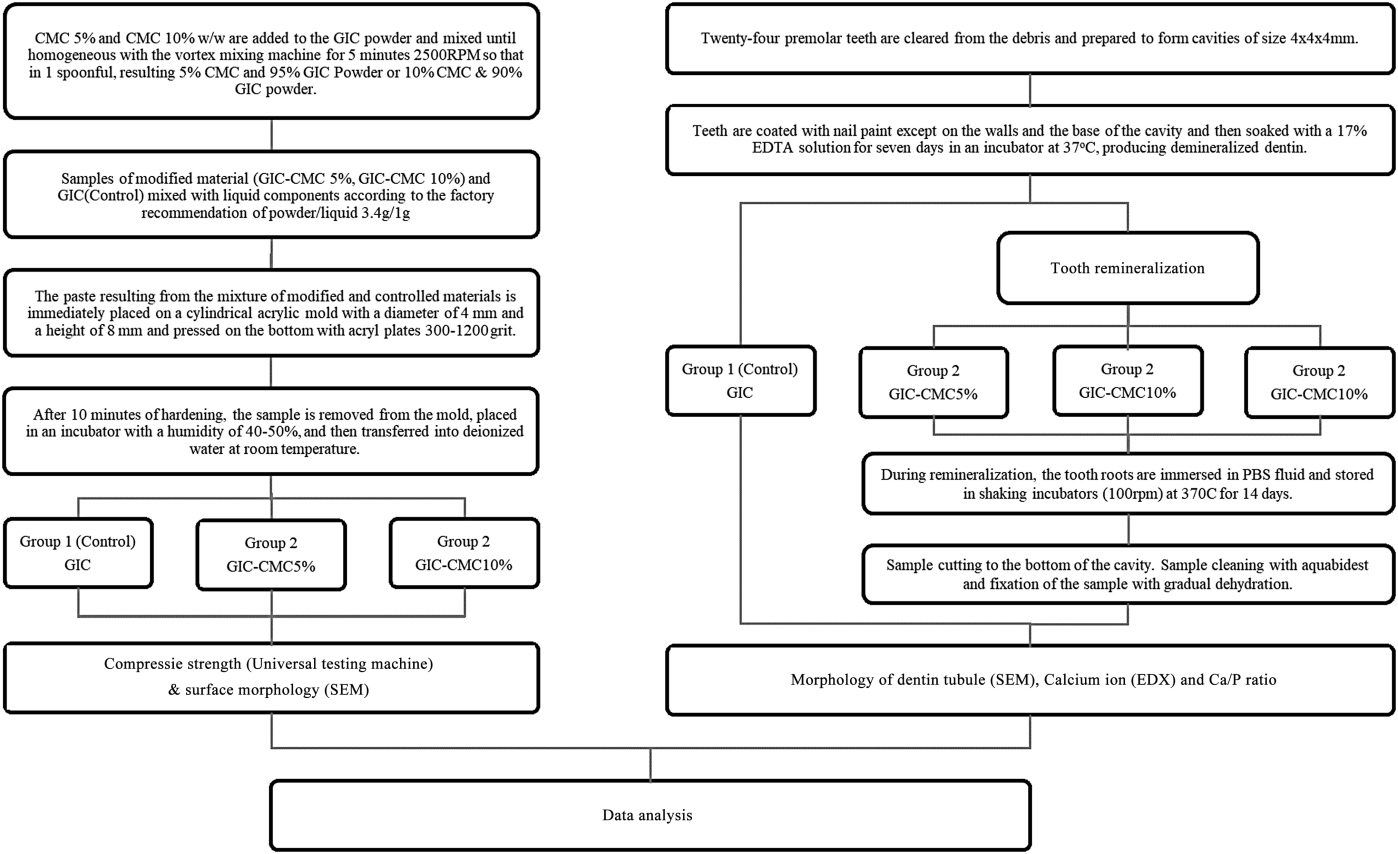
Research procedure flowchart. Abbreviations: EDTA, ethylenediaminetetraacetic acid; GIC-CMC, glass ionomer cement-carboxymethyl chitosan; SEM, scanning electron microscopy.

This study is a laboratory experimental test performed at the laboratory in Universitas Indonesia and the National Innovation Research Agency in February–May 2023 with clearance from the committee (Nomor: 7/Ethical Exempted/FKGUI/IX/2021 and Nomor: 11/Ethical Exempted/FKGUI/III/2023). This study uses a research procedure that can be seen in Fig. 1 . The samples in this study were human premolars with the criteria of the absence of caries, fillings, or root-crown defects and never undergoing orthodontic treatment. The human premolars were extracted for orthodontic treatment with a maximum storage period of 14 days. The research tools used were 100 mL bottles, diamond burs, high-speed handpieces, periodontal probes, digital scales, mixing slabs, plastic cement spatula, plastic filling, cement plugger, dental loupe, vortex spinning machine, shaking incubator, SEM, universal testing machine, and EDX machine. The research materials used were tooth samples according to the criteria, phosphate-buffered saline (PBS) solution, nail polish (Nail Enamel, Revlon, Oxford-North Carolina, United States), 17% ethylenediamine tetraacetic acid (EDTA) solution (OneMed, Indonesia), aquabidest, NaCl, alcohol concentrations of 50, 70, 80, 90, and 96%, CMC powder (PUI Chitosan and Advanced Materials, University of North Sumatra), and GIC (Fuji IX, GC Corp., Japan). The composition of the materials used in this study is summarized in Table 1 .

**Table 1:** Composition of materials

Material	Batch no.	Composition
GC Fuji IX GIC	N219047	Polyacrylic acid liquid and powder containing silica, calcium fluoride, and alumina
CMC Powder (PUI Chitosan and Advanced Materials, University of North Sumatra)	–	Carboxymethyl chitosan powder
Onemed Ethylenediaminetetraacetic acid 17%	12041675	Ethylenediaminetetraacetic acid 17%
Phosphate buffered saline pH 7.4	–	Phosphate buffered saline
Temp-It Light curing temporary restoration	18917923	Triethylene glycol dimethacrylate

Abbreviation **s** : CMC, carboxymethyl chitosan; GIC, glass ionomer cement.

CMC and GIC powder were placed on a 50 mL centrifugal tube and a vortex spinning machine to ensure the powder was mixed homogeneously before mixing with the GIC liquid provided in the FUJI IX product (GC Corp). The mixing of the GIC-CMC5% group was done by adding 0.17 g (5%) of CMC powder to the FUJI IX (GC Corp) GIC powder component weighing 3.23 g (95%). The mixing of the GIC-CMC10% group was done by adding 0.34 g (10%) of CMC powder to the FUJI IX (GC Corp) GIC powder component weighing 3.06 g (90%).

Thirty samples of the cement materials in the form of cylindrical shapes, made using an acrylic mold with a hole of 4 mm diameter and 8 mm thickness, were tested for the compressive strength of the cement group using a universal testing machine (Tensilon RTG, 10 kN, A&D, Japan) at a crosshead speed of 0.75 mm/min. The 30 samples were divided into three groups, namely GIC, GIC-CMC5%, and GIC-CMC10%, containing 10 samples in each cement group.

Samples of 28 premolars that met the criteria were divided into four groups: the control group (demineralized dentin), group I (demineralized dentin applied with GIC), group II (demineralized dentin applied with GIC-CMC5%), and group III (demineralized dentin applied with GIC-CMC10%). The sample was prepared to form a cavity measuring 3 × 3 × 3 mm. The tooth root was cut 2 to 3 mm from the apical end to facilitate the PBS solution in the cavity to simulate normal teeth in a physiologic state. The samples were demineralized by soaking in 17% EDTA for 1 week. The GIC-CMC modified material was made by adding CMC powder to GIC at ratios of 5 and 10% to obtain GIC-CMC5% and GIC-CMC10% materials, respectively. The GIC, GIC-CMC5%, and GIC-CMC10% were then placed in the cavity for groups I, II, and III, respectively. During the remineralization process, the sample roots were soaked in a shaking incubator containing PBS solution for 14 days to simulate the conditions of the teeth in the oral cavity.

The samples were observed using SEM to examine the modified materials and the morphological surface of the dentin. A universal testing machine was used to measure the compressive strength of the cylindrical materials. EDX analysis was conducted to measure the calcium ion content in the dentin and the composition ratio of the calcium ions compared with the phosphorus ions. The qualitative data on the surface morphology of dentin after the application of the materials and the surface of the materials itself were presented descriptively with images at 1,000x, 2,500x, and 5,000x magnification using SEM imaging software. Then, to support the descriptive analysis of SEM images, Image J (NIH, Bethesda, Maryland, United States) is used to analyze SEM images to obtain measurement values for semiqualitative analysis. The quantitative data on the calcium ions present in the dentin and compressive strength were analyzed statistically. Data normality was checked using the Shapiro–Wilk test, as the sample size was less than 50. If the data had a normal distribution, a one-way analysis of variance (ANOVA) with a significance level (α) less than 0.05 was used, followed by a post-hoc test using the Bonferroni test for homogeneous data. The Kruskal-Wallis test with a significance level (α) less than 0.05 was used for non-normally distributed data, followed by a Mann–Whitney U tests.

## Results

This study aimed to investigate the effects of modifying GIC with CMC on its surface morphology after applying the modified GIC-CMC5% and GIC-CMC10% on demineralized dentin in terms of changes in dentin surface morphology and calcium ion composition.

**Fig. 2:**
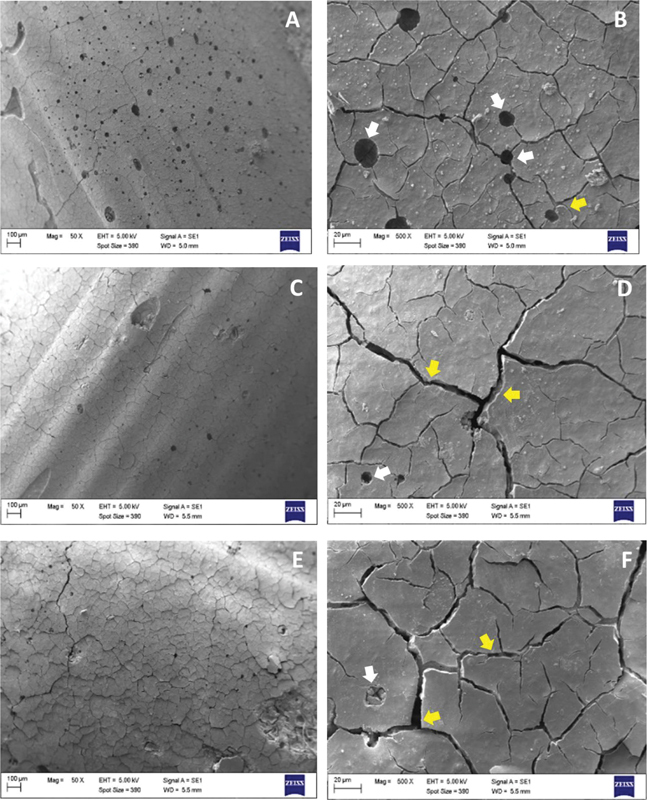
Surface morphology of modified GIC materials observed using SEM. ( **A** ) and ( **B** ) GIC group (control) with magnifications of 50x and 500x. ( **C** ) and ( **D** ) GIC-CMC5% modification group with magnifications of 50x and 500x. ( **E** ) and ( **F** ) GIC-CMC10% modification group with magnifications of 50x and 500x. The white arrows indicate pores, and the yellow arrows indicate cracks.

The SEM observation shows the surface morphology of the modified materials. As illustrated in Fig. 2 (A–B) GIC, **(C–D)** GIC-CMC5%, and **(E–F)** GIC-CMC10%, the SEM images showed a change in the surface morphology characterized by a tendency toward reduced porosity, and an increased number of cracks on the surface of the modified materials. The morphology of the GIC-CMC5% modification material showed reduced porosity and an increased number of surface cracks compared with that of GIC. In the GIC-CMC10% modification material, there was a decrease in porosity and a higher number of wider surface cracks compared with GIC and the GIC-CMC5% modification material.

**Fig. 3:**
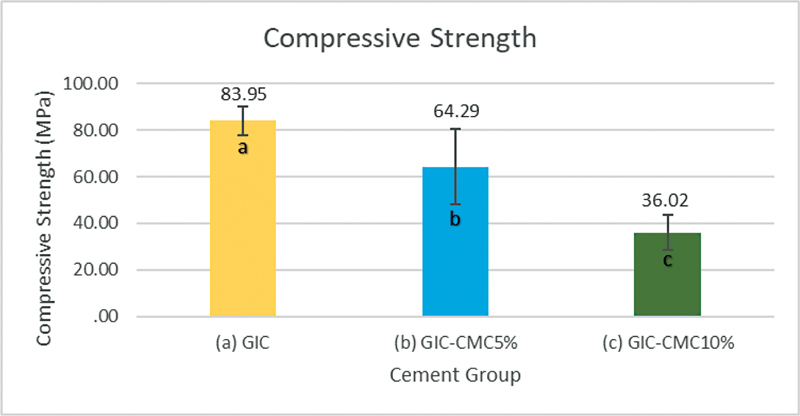
Compressive strength of GIC, GIC-CMC5%, and GIC-CMC10%. The error bars represent the standard deviation. Bars with different letters are statistically significant ( *n* = 10; *p* < 0.05, Tukey-HSD).

Fig. 3 shows the average compressive strength of the GIC-CMC5% and GIC-CMC10% modified materials. The highest average compressive strength was found in the GIC group (control) at 83.95 MPa, whereas the lowest average compressive strength was observed in the GIC-CMC10% group at 36.02 MPa. The one-way ANOVA showed a result of *p* = 0.000 ( *p* < 0.05), indicating a statistically significant difference among the compressive strength values of the three treatment groups.

The post-hoc Bonferroni test was used to determine that group showed differences in compressive strength values. The results of the test are presented in bars with different letters in Fig. 3 , which show significant differences in the mean compressive strength among the GIC-CMC5%, GIC-CMC10%, and GIC groups.

A significant difference of *p* = 0.001 ( *p* < 0.5) and a mean difference of 19.65 MPa were observed in the comparison between the GIC-CMC5% modified material and GIC (control). Similarly, a significant difference of *p* = 0.000 ( *p* < 0.5) and a mean difference of 47.92 MPa were found in the comparison between the GIC-CMC10% modified material and GIC. A significant difference of *p* = 0.000 ( *p* < 0.5) and a mean difference of 28.27 MPa were observed in the comparison between the GIC-CMC5% and GIC-CMC10% modified materials.

**Fig. 4:**
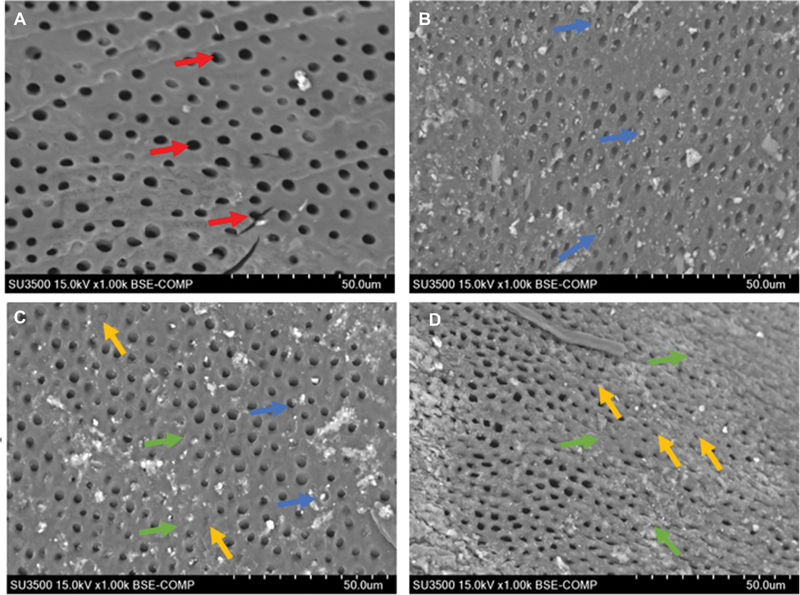
The dentin surface morphology was evaluated using scanning electron microscopy. ( **A** ) Demineralized dentin control group; ( **B** ) GIC group; ( **C** ) glass ionomer cement-carboxymethyl chitosan (GIC-CMC5%) group; ( **D** ) GIC-CMC10% group. The red arrows indicate an enlargement of dentin tubule size. The yellow arrows indicate a narrowing of dentin tubule size. The blue arrows indicate crystal occlusion. The green arrows indicate the thickening of the intertubular layer.

The samples for dentin remineralization were divided into four groups: demineralized dentin group, demineralized dentin group treated with GIC, demineralized dentin group treated with GIC-CMC5%, and demineralized dentin group treated with GIC-CMC10%. The results of the SEM analysis revealed differences in the surface morphology of demineralized dentin following the various treatments. Fig. 4A shows the typical characteristics of demineralized dentin, with numerous widely open dentin tubules and damage to the tubule edges due to demineralization. Fig. 4B shows crystal occlusion within the tubules and intertubular dentin after the application of GIC for 14 days, although no significant improvement was observed in the dentin tubule edges. Fig. 4C reveals that some dentin tubules underwent remineralization and narrowing following treatment with GIC-CMC5%, with thickening of the intertubular dentin layer. Fig. 4D illustrates that the treatment with GIC-CMC10% resulted in the most significant change, with improvements in the dentin tubule diameter, minimal crystal occlusion, and the highest thickening of the intertubular dentin layer. This was also confirmed through measurements in Image J (NIH, Bethesda, MD, USA), as shown in Table 1 . According measurement analysis ( Table 2 ) using *ImageJ* (NIH, Bethesda, Maryland, United States), the size of tubule diameter for GIC-CMC10% was 11.11 ± 3.91 µm compared with GIC-CMC5% (23.66 ± 3.13 µm), GIC (26.13 ± 8.48 µm), and demineralized dentin (40.87 ± 3.13 µm).

**Table 2:** Results of the mean dentin tubule's diameter size for each test group in micron

Group	Mean (SD)
Demineralized dentin	40.871 (3.13)
GIC	26.13 (8.48)
GIC-CMC5%	23.66 (3.13)
GIC-CMC10%	11.11 (3.91)

Abbreviations: GIC-CMC, glass ionomer cement-carboxymethyl chitosan; SD, standard deviation.

**Fig. 5:**
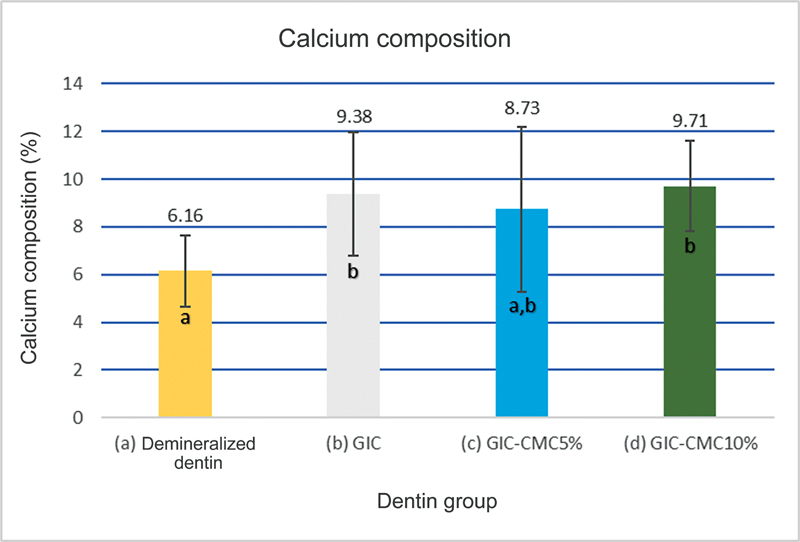
Calcium composition of demineralized dentin, GIC, glass ionomer cement-carboxymethyl chitosan (GIC-CMC5%), and GIC-CMC10%. The error bars represent the standard deviation. Bars with different letters are statistically significant ( *n* = 10; *p* < 0.05, Tukey-honest significant difference).

In this study, EDX was used to assess the calcium ion levels in the samples and to confirm the formation of HAP in the dentin. The results are presented in Fig. 5 , which showed that the demineralized dentin control group had the lowest calcium levels (6.16%), while the application of GIC, GIC-CMC5%, and GIC-CMC10% materials increased the calcium ion levels, with the GIC-CMC10% group exhibiting the highest level (9.71%). Statistical analysis using the Kruskal–Wallis test indicated significant differences in the calcium ion level among the groups ( *p* < 0.05). The post-hoc Mann–Whitney U tests ( Fig. 5 ) revealed significant differences between the demineralized dentin control group and the GIC and GIC-CMC10% groups, but no significant difference was observed between the GIC-CMC5% and GIC-CMC10% groups. These results suggest that the application of GIC-CMC10% had the most significant effect on the calcium ion levels in demineralized dentin during the remineralization process over 14 days, while the application of GIC-CMC5% did not show a significant effect.

The EDX results for the demineralized dentin control group indicated the lowest Ca/P ratio of 0.91 compared with the other groups ( Table 3 ). After the application of GIC and GIC modified with 5 and 10% CMC (GIC-CMC) materials for 14 days, the Ca/P ion ratio increased. The GIC-CMC10% group showed the highest Ca/P ion ratio (1.58), almost reaching the Ca/P ion ratio of HAP (1.67). The Kruskal–Wallis statistical test did not indicate significant differences between the groups from a substantive perspective. The increase in the Ca/P ion ratio in the GIC-CMC10% group suggests an increase in remineralization success, nearing the Ca/P ion ratio of HAP.

**Table 3:** Results of the mean Ca/P ion ratio comparison for each test group

Group	Mean (SD)	*p* -Value
Demineralized dentin	0.91 (0.62)	0.319
GIC	0.93 (0.28)	
GIC-CMC5%	1.03 (0.41)	
GIC-CMC10%	1.58 (0.58)	

Abbreviations: Ca/P, calcium-to-phosphorous; GIC-CMC, glass ionomer cement-carboxymethyl chitosan; SD, standard deviation.

## Discussion

The selection of modified GIC materials with CMC concentrations of 5 and 10% was based on research by Mulder and Anderson-Small, who discovered that this modification could be performed successfully because the hydroxyl groups of chitosan can bond with silicon ions in GIC during the setting reaction and form strong silanol bonds. 15 CMC's cationic CH amino group also interacts electrostatically with the carboxylic acid group in GIC liquid. 15 17 It forms various polyelectrolyte complexes, which increase the release of ions such as aluminum, strontium, silicon, and sodium, which can improve the physical properties of GIC. 15 17 Mixing with this composition is also known not to reduce the compressive strength, solubility, and hardening time of the GIC due to its unmodified powder composition of more than 10%, so the amount of fillers contained is still sufficient to maintain the physical properties of GIC. 17 Research by Mulder and Anderson-Small showed that a decrease in the physical properties of GIC would occur if the powder composition changed by up to 20%. 15 17

The pores in GIC are generally caused by the entrapment of air during the mixing procedure. The effects of these pores have not been fully understood. However, it is assumed that they affect the strength of GIC. 18 As shown in Fig. 2 , the SEM results of the surface morphology of the materials show a reduction in the number of pores and an increase in the number of wider cracks with the addition of 5% CMC and 10% CMC. The formation of an increasing number of wider cracks can be associated with a reduction in the compressive strength of the GIC-CMC5% and GIC-CMC10% modified materials ( Fig. 2 ). 19 This likely occurs because the excessive increase in the CMC mass percentage makes some CMC chains prone to agglomeration rather than interacting with inorganic particles and the polycarboxylate matrix of GIC. It is suspected that the reduction in porosity is due to the agglomeration of CMC, which disrupts the integrity of the GIC structure, thereby affecting the compressive strength of the modified material. 12 20 Panpisut et al observed various powder–liquid ratios of modified GIC with pre-reacted spherical glass filler in terms of setting time, fluoride release, and compressive strength and found that a powder–liquid ratio greater than 1.89 was required to achieve compressive strength exceeding 100 MPa. However, increasing the powder–liquid ratio to 3:1 led to a decrease in compressive strength due to difficulties in the mixing phase, resulting in a reduced acid–base neutralization reaction, thereby causing a reduction in polymer cross-linking. 17 21 22 It is possible that this could be related to this study, in which the modification of GIC with the addition of 5% or 10% CMC in the powder component of GIC affects the powder–liquid ratio, consequently decreasing the compressive strength of the tested material ( Fig. 3 ). Thus, the first and second hypotheses have to be accepted: the increased concentration of CMC in GIC affects the surface morphology and the compressive strength of the materials.

Treatment of demineralization in dentin using 17% EDTA for 7 days in this study is consistent with what was done by Chen et al, Annisa et al, Yamin et al, and Maharti et al. 5 23 24 25 This technique was applied because it is a potent chelating agent for dentin and to get a cavity simulation resembling the dentin-affected condition, simulate mineral-depleted dentin surfaces, and rarely leaves minerals on the surface of dentin. 5 23 24 25 26 The 6-day use of EDTA for dentin demineralization applied by Carvalho et al, who stated that the fibril structure of collagen did not occur degradation and denaturation through transmission electron microscopy (TEM) examination. 27 Annisa et al also stated that EDTA can remove all minerals in dentin while maintaining an intact collagen structure. 23

This study demonstrates that the application of GIC-CMC, particularly GIC-CMC10%, in demineralized dentin for over 14 days could stimulate remineralization changes in the dentin surface morphology and increase calcium ion levels, which play a crucial role in the remineralization process. The exposure of remineralization materials for 14 days has been described by Maharti et al and Setiati et al. 16 25 Setiati et al also stated the use of 10% of CMC-ACP can achieve the highest dentin remineralization after 14 days of exposure. 16 Maharti et al stated that the application of 5% CMC/ACP-gypsum mixture shows denser mineral deposits with more irregular edges of dentin tubules. 25 The GIC-CMC10% group was successful in forming crystals that closely resembled HAP in demineralized dentin, indicating the potential for biomimetic remineralization to enhance the strength and resistance to caries of the affected dentin. 28 The SEM observations confirmed that the GIC-CMC10% treatment group achieved the optimal remineralization results ( Fig. 4 ), with the smallest dentin tubule size ( Table 2 ) indicating the thickest intertubular dentin layer compared with the other groups, and minimal crystal occlusions on the dentin surface. According to this analysis, the third hypothesis, that the increased concentration of CMC in GIC affects the surface morphology of the dentin tubule, has to be accepted.

In the demineralized dentin control group, wide-open dentin tubules showed a demineralization process using EDTA 17%. The GIC group exhibited numerous crystal occlusions on the dentin surface but failed to restore the dentin tubule structure, which resulted from the demineralization process. The character of GIC when binding to tooth tissue is the presence of a mechanism of hydrogen binding between the carboxyl group of the polyacid and calcium in the enamel and dentin. The fluoride content during the acid–base reaction does not inhibit caries due to the formation of fluorohydroxyapatite on the tooth bond, which causes the area to be more resistant to the demineralization process. 29 However, in nanotechnology terminology, such mechanisms are classified as top–down remineralization or partial remineralization. 30 This process also results in hypermineralization on the surface of the lesion, thus inhibiting remineralization in the deeper parts. 31 The remineralization ability of the GIC-CMC5% group was better than that of the GIC group but was lower compare with the GIC-CMC10% group. These SEM findings are in accordance with those of Chen et al and Budiraharjo et al, which showed that modifying MTA and ACP with CMC led to increased mineral precipitation and the repair of demineralized dentin tubules compared with unmodified materials. 5 6 This study also found that biomimetic remineralization occurred, filling both intrafibrillar and extrafibrillar dentin spaces. These similar results suggest that remineralization in this study is also an effective biomimetic remineralization. Support for these findings can be found in previous research in which the GIC-CMC10% group exhibited the ability to stabilize calcium ions in amorphous form, allowing them to enter the intrafibrillar collagen of dentin and facilitating biomimetic remineralization. 5 8

As an analytical technique, EDX helped confirm the observed significant increase in calcium ion levels in the GIC-CMC10% group and substantiated the measurement results ( Fig. 5 ). The composition results of calcium ions in the GIC-CMC5% group showed an increase compared with the demineralized dentin control group after a 14-day remineralization process. However, this difference was not statistically significant. The comparison of increased calcium ion levels between the GIC-CMC10% and GIC-CMC5% groups did not exhibit statistically significant differences. These outcomes are in accordance with this study of Mulder and Anderson-Small, which demonstrated that ions such as strontium did not undergo significant changes when GIC was modified with 5% chitosan but exhibited significant changes when GIC was modified with 10% chitosan. 15 The increase in ion release occurs due to the mixing of chitosan, which binds various ions into its hydroxyl groups through ionic interactions and rapidly reacts with the liquid component of GIC. 15 Consequently, these ions are not bound in the initial stages of GIC hardening and can be released when the GIC absorbs water. 15 The same results were found in Budiraharjo et al.'s study, in which the modification of MTA with CMC demonstrated a higher increase in calcium ions compared with the control group. 6 Thus, the fourth hypothesis that the increased concentration of CMC in GIC increases calcium ion has to be rejected.

Although the differences in the Ca/P ion ratio results in the treatment groups did not show significant statistical differences ( Table 3 ) from a substantive perspective, the increase in the Ca/P ion ratio in the GIC-CMC10% group ( Table 3 ) suggests the formation of HAP crystals that were closer to the ideal form (Ca/ *p* = 1.67). 23 32 33 The results of this study support previous findings indicating that CMC plays a significant role in dentin remineralization. By stabilizing ACP nano precursors, CMC inhibits their aggregation into HAP before entering the intrafibrillar collagen of dentin, thus optimizing the formation of denser and more stable HAP crystals. 6 23 The high Ca/P ion ratio in the GIC-CMC10% group ( Table 3 ) suggests the formation of calcium-deficient HAP crystals with the potential to mature into HAP, enhancing the physical properties of dentin, including elasticity modulus and resistance to caries. 34 35 Different results were observed in the Ca/P ion ratio between the GIC group and the GIC-CMC5% group, indicating a lack of crystal maturation toward hydroxyapatite. 36 The low ratio results occurred due to the insufficient supply of calcium ions by the GIC and GIC-CMC5% as the raw materials for the remineralization process. 6 8 37 Thus, the fifth hypothesis that the increased concentration of CMC in GIC increases Ca/P ratio has to be accepted.

Biomimetic remineralization using GIC-CMC materials offers the potential to enhance the natural physical properties and caries resistance of demineralized dentin. 38 Although this study was conducted *in vitro* using extracted premolar teeth, it provides a basis for further research that may involve *in vivo* and clinical studies to validate the success of biomimetic remineralization in patients with caries-affected teeth. Future research may explore the potential of biomimetic remineralization of GIC-CMC modified materials in the intrafibrillar or gap zone collagen of demineralized dentin using TEM analysis. With TEM, changes in the internal structure of the dentin can be examined in more detail to assess mineral deposition in the intrafibrillar dentin space, which plays a critical role in improving the mechanical strength and caries resistance of the dentin. In addition, *in vivo* research can be conducted to observe the clinical success of remineralization and to compare the results with this *in vitro* study. 39

The use of GIC-CMC10% optimizes the remineralization process and forms HAP crystals that closely resemble the ideal form of demineralized dentin. These results suggest the clinical application potential of enhancing the physical properties and caries resistance of demineralized dentin. 40 Continued research and development in this field are expected to discover more effective biomimetic remineralization materials and to provide an alternative for the treatment of demineralized dentin caused by caries. In addition, in future studies, research could be undertaken to calculate the estimates of fluoride ions and their role in remineralization. Then, the tensile strength test is needed to complete the compressive strength test and improve understanding of the GIC-CMC modified material.

## Limitations

The limitations of this study include the inability to evaluate mineral deposition within intrafibrillar dentin and the need for more research regarding successful clinical application. Testing with TEM devices and future *in vivo* studies may address these limitations.

## Conclusion

This study found that the surface morphology of GIC modified with the addition of CMC tended to show a reduction in porosity, an increase in the area of wider cracks, and a decrease in compressive strength of the material with the increasing percentage of CMC. GIC modified with 10% CMC affected the changes in the morphology and calcium ion composition of demineralized dentin with crystal formation similar to HAP.

## References

[1] Niu L N Zhang W Pashley D H Biomimetic remineralization of dentin Dent Mater 2014 30 01 77 96 23927881 10.1016/j.dental.2013.07.013 PMC3867526

[2] Bacino M Girn V Nurrohman H Integrating the PILP-mineralization process into a restorative dental treatment Dent Mater 2019 35 01 53 63 30545611 10.1016/j.dental.2018.11.030 PMC6312741

[3] He L Hao Y Zhen L Biomineralization of dentin J Struct Biol 2019 207 02 115 122 31153927 10.1016/j.jsb.2019.05.010

[4] Hernández M Cobb D Swift E J Jr Current strategies in dentin remineralization J Esthet Restor Dent 2014 26 02 139 145 24612484 10.1111/jerd.12095

[5] Chen Z Cao S Wang H Biomimetic remineralization of demineralized dentine using scaffold of CMC/ACP nanocomplexes in an in vitro tooth model of deep caries PLoS One 2015 10 01 e0116553 25587986 10.1371/journal.pone.0116553 PMC4294661

[6] Budiraharjo R Neoh K G Kang E T Kishen A Bioactivity of novel carboxymethyl chitosan scaffold incorporating MTA in a tooth model Int Endod J 2010 43 10 930 939 20738427 10.1111/j.1365-2591.2010.01771.x

[7] Shariatinia Z Carboxymethyl chitosan: properties and biomedical applications Int J Biol Macromol 2018 120(Pt B):1406 1419 30267813 10.1016/j.ijbiomac.2018.09.131

[8] Putranto A W Suprastiwi E Meidyawati R Agusnar H Characterization of novel cement-based carboxymethyl chitosan/amorphous calcium phosphate Eur J Dent 2022 16 04 809 814 35016237 10.1055/s-0041-1739449 PMC9683891

[9] Gao W Smales R J Yip H K Demineralisation and remineralisation of dentine caries, and the role of glass-ionomer cements Int Dent J 2000 50 01 51 56 10945181 10.1111/j.1875-595x.2000.tb00547.x

[10] Moheet I A Luddin N Rahman I A Kannan T P Nik Abd Ghani N R Masudi S M Modifications of glass ionomer cement powder by addition of recently fabricated nano-fillers and their effect on the properties: a review Eur J Dent 2019 13 03 470 477 31280484 10.1055/s-0039-1693524 PMC6890502

[11] Arjomand M E Eghlim M H Jalalian S H Mirzakhani M Mahavi A Effects of aging on compressive strength of two resin-reinforced glass ionomers: an in-vitro study J Res Dent Maxillofac Sci 2019 4 03 15 20

[12] Bao X Liu F He J Preparation and characterization of glass ionomer cements with added carboxymethyl chitosan J Macromol Sci Part B Phys 2020 59 06 345 356

[13] Kashyap P K Chauhan S Negi Y S Goel N K Rattan S Biocompatible carboxymethyl chitosan-modified glass ionomer cement with enhanced mechanical and anti-bacterial properties Int J Biol Macromol 2022 223(Pt A):1506 1520 36368362 10.1016/j.ijbiomac.2022.11.028

[14] Pratiwi D Salim R F Tjandrawinata R Komariah K Evaluasi morfologi permukaan semen ionomer kaca dengan modifikasi penambahan nanokitosan kumbang tanduk Surface morphology evaluation of glass ionomer cement modified with nano chitosan of rhinoceros beetle J Kedokt Gigi Univ Padjadjaran 2021 33 03 240

[15] Mulder R Anderson-Small C Ion release of chitosan and nanodiamond modified glass ionomer restorative cements Clin Cosmet Investig Dent 2019 11 313 320 10.2147/CCIDE.S220089 PMC6737163 31686917

[16] Setiati H D Suprastiwi E Artiningsih D ANP Utami L PTB Concentration dependent effects of carboxymethyl chitosan on dentin remineralization with amorphous calcium phosphate Int J Appl Pharm 2020 12 02 31 33

[17] Mulder R Variation in the dispersions of powder liquid ratios in hand-mix glass ionomers Open Dent J 2018 12 01 647 654 30369974 10.2174/1745017901814010647 PMC6182876

[18] Nicholson J W The History and Background to Glass-Ionomer Dental Cements Springer Cham 2016 10.1007/978-3-319-22626-2_1

[19] Sidhu S K Nicholson J W A review of glass-ionomer cements for clinical dentistry J Funct Biomater 2016 7 03 16 27367737 10.3390/jfb7030016 PMC5040989

[20] Soygun K Soygun A Dogan M C The effects of chitosan addition to glass ionomer cement on microhardness and surface roughness J Appl Biomater Funct Mater 2021 19 2.280800021989706E15 10.1177/2280800021989706 33784189

[21] Panpisut P Monmaturapoj N Srion A Angkananuwat C Krajangta N Panthumvanit P The effect of powder to liquid ratio on physical properties and fluoride release of glass ionomer cements containing pre-reacted spherical glass fillers Dent Mater J 2020 39 04 563 570 32037386 10.4012/dmj.2019-097

[22] Fleming G JP Farooq A A Barralet J E Influence of powder/liquid mixing ratio on the performance of a restorative glass-ionomer dental cement Biomaterials 2003 24 23 4173 4179 12853247 10.1016/s0142-9612(03)00301-6

[23] Annisa R N Djauharie N Suprastiwi E Avanti N The effect of carboxymethyl chitosan/amorphous calcium phosphate to guide tissue remineralization of dentin collagen Int J Appl Pharm 2019 11 01 181 183

[24] Yamin E Suprastiwi E Usman M Sarmayana S The effect of gypsum extension on a mixture of carboxymethyl chitosan and amorphous calcium phosphate in dental remineralization J Stomatol 2020 73 02 69 73

[25] Maharti I D Suprastiwi E Setiati H D Yamin E Cahyani A N The effects of mixtures of various concentrations of carboxymethyl chitosan/amorphous calcium phosphate with gypsum on dentin remineralization Int J Appl Pharm 2020 12 02 13 15

[26] Kim H J Bae H E Lee J E Effects of bioactive glass incorporation into glass ionomer cement on demineralized dentin Sci Rep 2021 11 01 7016 33782472 10.1038/s41598-021-86481-y PMC8007704

[27] Carvalho R M Tay F Sano H Yoshiyama M Pashley D H Long-term mechanical properties of EDTA-demineralized dentin matrix J Adhes Dent 2000 2 03 193 199 11317392

[28] Sereda G VanLaecken A Turner J A Monitoring demineralization and remineralization of human dentin by characterization of its structure with resonance-enhanced AFM-IR chemical mapping, nanoindentation, and SEM Dent Mater 2019 35 04 617 626 30808558 10.1016/j.dental.2019.02.007

[29] Weiner R Liners and bases in general dentistry Aust Dent J 2011 56 01 11 22 21564112 10.1111/j.1834-7819.2010.01292.x

[30] Niu L N Zhang W Pashley D H Biomimetic remineralization of dentin Dent Mater 2014 30 01 77 96 23927881 10.1016/j.dental.2013.07.013 PMC3867526

[31] Abduo J Swain M Self-reparability of glass-ionomer cements: an in vitro investigation Eur J Oral Sci 2011 119 02 187 191 21410561 10.1111/j.1600-0722.2011.00810.x

[32] Maharti I D Suprastiwi E Setiati H D Yamin E Cahyani A N The effects of mixtures of various concentrations of carboxymethyl chitosan/amorphous calcium phosphate with gypsum on dentin remineralization Int J Appl Pharm 2020 12 02 13 15

[33] Tariq U Haider Z Chaudhary K Hussain R Ali J Calcium to phosphate ratio measurements in calcium phosphates using LIBS J Phys Conf Ser 2018 1027 12015 10.1088/1742-6596/1027/1/012015

[34] Hu Y Wan L Xiao Y Enhanced reparative dentinogenesis of biphasic calcium phosphate ceramics containing calcium-deficient hydroxyapatite (CDHA) and strontium-incorporated CDHA in direct pulp capping Mater Today Commun 2022 33 104231

[35] Moseke C Gbureck U Tetracalcium phosphate: Synthesis, properties and biomedical applications Acta Biomater 2010 6 10 3815 3823 20438869 10.1016/j.actbio.2010.04.020

[36] Lodoso-Torrecilla I Klein Gunnewiek R Grosfeld E-C Bioinorganic supplementation of calcium phosphate-based bone substitutes to improve in vivo performance: a systematic review and meta-analysis of animal studies Biomater Sci 2020 8 17 4792 4809 32729591 10.1039/d0bm00599a

[37] Kim Y K Yiu C KY Kim J R Failure of a glass ionomer to remineralize apatite-depleted dentin J Dent Res 2010 89 03 230 235 20110510 10.1177/0022034509357172 PMC2826886

[38] Cheah C W Al-Namnam N M Lau M N Synthetic material for bone, periodontal, and dental tissue regeneration: where are we now, and where are we heading next? Materials (Basel) 2021 14 20 6123 34683712 10.3390/ma14206123 PMC8537464

[39] Lynch R JM Mony U ten Cate J M Effect of lesion characteristics and mineralizing solution type on enamel remineralization in vitro Caries Res 2007 41 04 257 262 17556834 10.1159/000101914

[40] Bertassoni L E Habelitz S Kinney J H Marshall S J Marshall G W Jr Biomechanical perspective on the remineralization of dentin Caries Res 2009 43 01 70 77 19208991 10.1159/000201593 PMC2698028

